# microRNAs and oligometastasis

**DOI:** 10.18632/aging.100731

**Published:** 2015-03-25

**Authors:** Nikolai N. Khodarev, Sean P. Pitroda, Ralph R. Weichselbaum

**Affiliations:** The University of Chicago, Chicago, IL, 60637, USA

Most cancer-related deaths occur as a result of metastases – tumor cells derived from the primary tumor which colonize distant sites. Metastases widely differ in their behavior which contributes to the heterogeneity in clinical manifestations of metastatic disease. To highlight this heterogeneity the concept of “oligometastases” was introduced two decades ago (Hellman and Weichselbaum. J Clin Oncol. 1995;13:8–10). The oligometastatic hypothesis posits the existence of a state of limited metastatic disease in contrast to the widely held notion that metastasis is a widespread disease (i.e. polymetastatic). Patients with oligo-metastatic cancers could potentially be cured with localized, metastasis-directed therapies, such as surgery and radiotherapy, while most metastatic patients are incurable even with systemic therapies. Two central questions arise: (1) are there molecular differences between oligometastases and polymetastases and (2) can we predict the behavior of metastatic disease in a given patient? The answers to these questions would advance our understanding of metastasis development and potentially lead to the discovery of biomarkers guiding the appropriate selection of therapeutic modalities for individual patients.

Recently we collected clinical samples derived from cancer patients with oligo- or poly-metastatic disease based on the number of metastatic lesions and their rate of metastatic progression [1,2]. We profiled microRNAs (miRs) from patient-derived samples to examine their potential roles in gene regulation mediating the oligometastatic phenotype. We investigated two cohorts of patients. In the first cohort, patients were treated with stereotactic body radiotherapy (SBRT) to all sites of metastatic disease [1]. In the second cohort, patients underwent surgical removal of lung metastases from various primary tumors [2]. In both cohorts, oligometastatic patients demonstrated significantly longer overall and disease-free survival as compared to polymetastatic patients. Together, 112 samples from both cohorts were profiled to identify differences in miR expression. Among these, 35 miRs and 15 miRs were differentially expressed in surgically resected lung oligometastases and SBRT treated oligometastases, respectively. We designated these as “oligomiRs” [3]. This finding provided evidence that oligometastases are a specific clinical entity and prompted a detailed examination into the molecular underpinnings of the actions of these miRs for their potential use as prognostic and predictive markers for subtypes of metastatic disease.

We mapped ~20,000 computer-predicted target genes to 101 specific KEGG pathways for the clinically identified oligomiRs. These pathways were enriched by cellular processes mediating adhesion, invasion and migration – the” AIM” phenotype [4]. We further found that ectopic expression of the oligomiRs 127-5p, 544a and 655-3p led to suppression of 1,376 to 2,134 individual mRNAs for each of the tested miRs. Target mRNAs mediate TGF-beta, RHO/ROCK and cadherin signaling linked to the AIM phenotype. We cloned the 3′ untranslated regions (UTR) of key genes from these pathways which validated their specific targeting by selected oligomiRs [5]. Using *in vivo* models of metastatic lung colonization we demonstrated that overexpression of oligomiRs or suppression of their target genes yielded a phenotype of limited metastatic development. These results convincingly demonstrated a causal relationship between oligomiR expression and the oligometastatic phenotype. Importantly, oligomiRs appeared to have no effect on the growth potential of metastatic cells suggesting that the AIM phenotype might be pivotal for successful lung colonization [4].

Analysis of the genomic distribution of oligomiRs revealed a surprising result in that a significant number of these miRs, including validated 127-5p, 544a and 655-3p miRs, are encoded in the 14q32 imprinted Dlk1-Dio3 locus of microRNAs. 14q32 chromosomal aberrations were first reported in 1983 in association with facial dysmorphism and mental retardation (J Med Genet. 1997; 34:515–517). The 14q32 cluster is also implicated in the pathogenesis of schizophrenia and alcoholism-associated syndromes [5]. In addition, the 14q32 locus was reported as critical in the early development of mouse embryos and associated with successful stem cell function leading to the generation of viable newborns [6]. Recent publications have also implicated the 14q32 locus in tumorigenesis [7]. Such a broad range of biological activities suggests a fundamental role in normal and pathologic developmental processes and stimulates questions regarding the contribution of 14q32-encoded miRs in the evolution of oligo- and poly-metastases. One interesting question is the role of epigenetic modification in oligometastasis development given the known role of DNA methylation in maintenance of the imprinted status of 14q32.

Taken together, our data suggest that oligometastases represent a unique clinical entity with distinct molecular features, one of which is characterized by differentially expressed miRs, or oligomiRs, and their effects on downstream target pathways in potentially complex interactions. Thus, the miR/gene networks are likely related to degree of metastasis rather than the usual binary endpoint used in experiments in which metastasis is either turned on or off. Secondly, a significant fraction of oligomiRs are encoded in the 14q32 imprinted chromosomal locus involved in many essential developmental processes. Involvement of this locus in the regulation of oligometastases emphasizes the fundamental nature of miR-dependent regulation of metastasis development and suggests parallels between the development of metastases in distant sites and embryonic development. Finally, these findings suggest that cellular growth properties may be dispensable in the regulation of metastasis development through reliance on regulation of the AIM phenotype – a result which calls for revision of our current understanding of therapeutic modalities for the treatment of metastatic disease.
